# Abdominal Wall Hydatid Cyst: Case Report and Review of Literature

**DOI:** 10.1155/2012/583294

**Published:** 2012-08-16

**Authors:** V. Abhishek, Vijayraj S. Patil, Ullikashi Mohan, B. S. Shivswamy

**Affiliations:** ^1^Department of General Surgery, Victoria Hospital, Bangalore Medical College and Research Institute, Bangalore 560002, India; ^2^Vijay Doctors Colony, Konanakunte Bangalore, Karnataka, Bangalore 560062, India

## Abstract

A 60-year-old woman presented with a swelling in right paraumbilical region of one-year duration. Examination revealed a painless cystic swelling 15 × 10 cm with cough impulse. Ultrasound and CECT abdomen showed a subcutaneous cystic swelling with intramuscular extension. No other intraabdominal cystic lesions were found. Surgical exploration showed a cystic lesion adherent to peritoneum without any intraperitoneal extension. Cyst was carefully excised without any spillage. Gross specimen on opening showed multiple daughter cysts consistent with hydatid cyst, confirming the diagnosis of solitary abdominal wall hydatid cyst.

## 1. Introduction

 Hydatid disease is a parasitic tapeworm infection that usually involves liver and lungs. Primary skeletal muscle hydatid cyst without liver and lung involvement is rare even in endemic districts. Muscular hydatosis has been well documented in literature but involvement of abdominal wall is a rare entity with about 5 cases reported till date. It is interesting to note that in all 5 cases the cyst location has been in right paraumbilical and iliac fossa. We report a case of abdominal wall hydatid with an attempt to explain the mechanism for this unusual location.

## 2. Case Report

**Figure 1:**
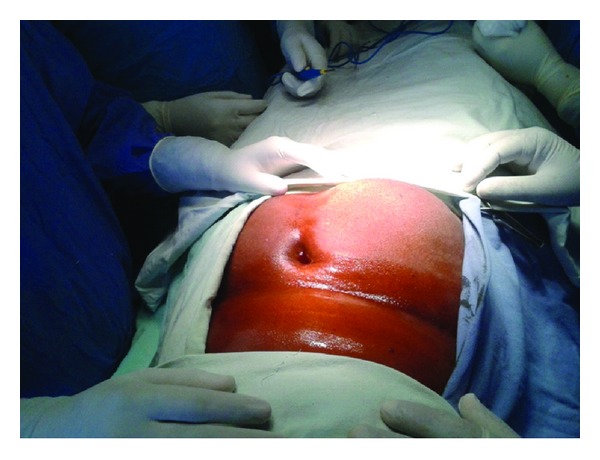
Preoperative swelling in right paraumbilical region.

**Figure 2:**
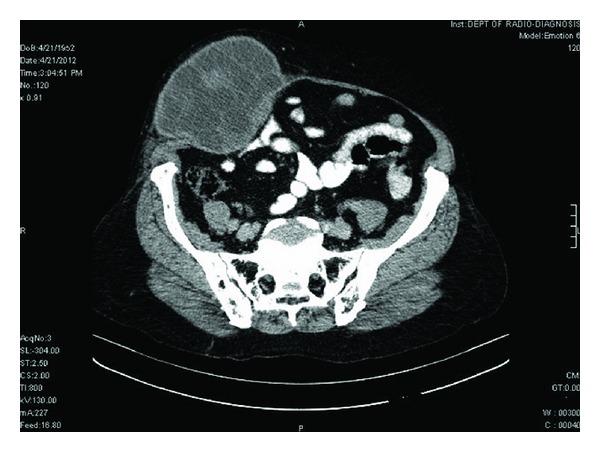
CECT showing large subcutaneous cyst with muscular extension and intact peritoneum.

**Figure 3:**
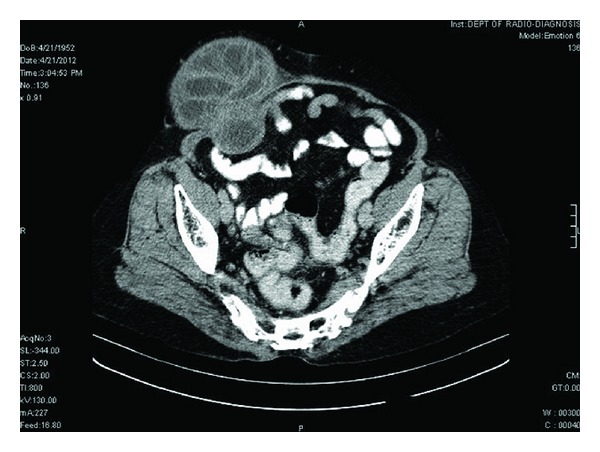
CECT Cyst with internal undulating septae.

**Figure 4:**
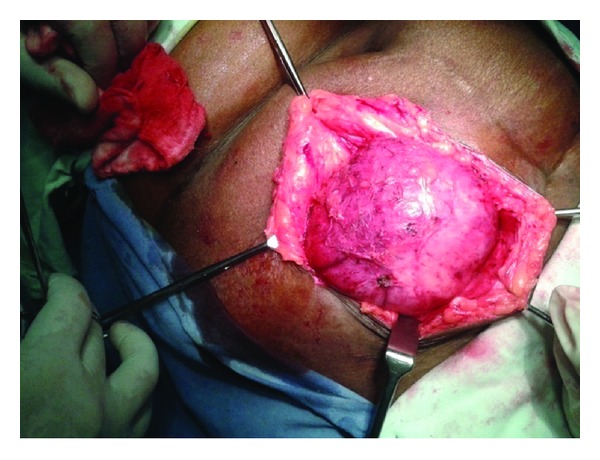
Intraoperative subcutaneous cyst.

**Figure 5:**
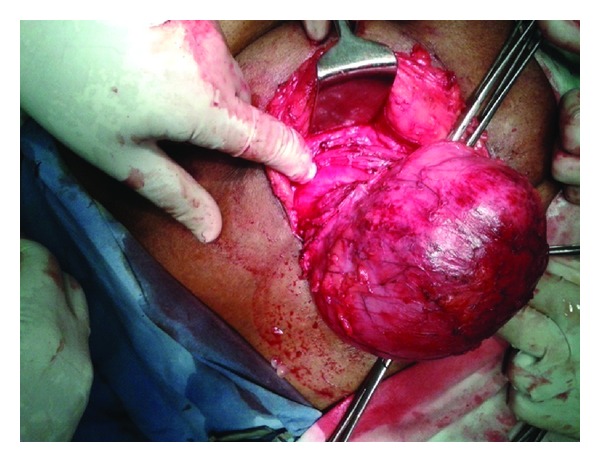
Intraoperative cyst with extension into abdominal wall muscle.

**Figure 6:**
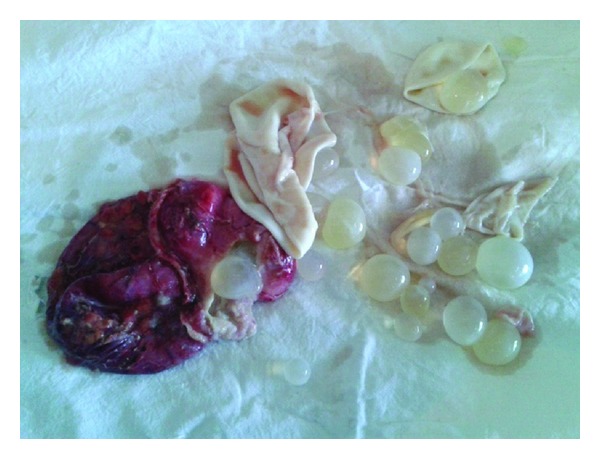
Specimen opened showing daughter cysts.

A 60-year-old woman presented with swelling in right paraumbilical region evolving over a year (Figure 1). Physical examination revealed a painless cystic swelling 15 × 10 cm with cough impulse positive. The overlying skin had dilated veins. A provisional diagnosis of paraumbilical hernia was made abdominal ultrasound showed a cystic lesion in subcutaneous and muscular plane with echogenic content within. Abdominal contrast enhanced computer tomography showed a predominantly subcutaneous cystic swelling with intra muscular extension without breach of peritoneum (Figure 2). The cyst showed internal undulating septae (Figure 3). No other intraabdominal cystic lesions were found. The preoperative examinations (chest radiograph, complete blood count, urine analysis, and blood biochemistry) revealed no abnormalities. A preoperative diagnosis of probable hydatid cyst was made and patient started on oral Albendazole. Surgical exploration revealed a cystic mass arising from parietal wall upon dissection the cyst wall was found adherent to peritoneum to prevent rupture of cyst a part of peritoneum was also excised along with cyst wall (Figures 4 and 5). The cyst showed no adhesions to omentum or any bowel. The cyst peritoneum was closed and abdominal wall defect closed with prolene mesh. The gross specimen on opening showed multiple daughter cysts and confirmed histopathologically as hydatid cyst (Figure 6). Postoperatively patient was put on combination of albendazole and Praziquantel for 3 months. Followup over six months showed no recurrence.

## 3. Discussion

Hydatidosis is a zoonotic infection caused by tapeworms belonging to the class Cestoda, in the family Taeniidae, of the genus Echinococcus. The *Echinococcus granulosus* species, which is responsible for cystic hydatidosis, has an almost ubiquitous diffusion. South America, Central Asia, and the Mediterranean basin [1] must be considered highly endemic areas.

The adult worm (3 to 6 mm long) lives in the small intestine of the definitive hosts, that is, dogs or other canids. Gravid proglottids containing infective eggs are shed daily through the faeces. After ingestion by a suitable intermediate host (usually herbivores like sheep, goats, swine, cattle, horses, camels, and occasionally also humans), the eggs hatch in the small intestine releasing a hooked larva called oncosphere. It penetrates the intestinal wall by means of its six hooks and migrates through the circulatory system reaching various organs, mainly the liver and lungs. Here, the oncosphere loses the hooks and develops into a cyst that enlarges gradually. Usually by the fifth month, the wall of the cyst differentiates into an outer laminated nonnucleated layer and an inner nucleated germinal layer. The inner layer produces protoscolices and daughter cysts that fill the cyst interior, which can be attached or floating free within the cyst fluid. The dog becomes infected after swallowing the cyst-containing organs of the slaughtered parasitized herbivores. The ingested protoscolices attach to the intestinal mucosa, and develop into adult stage tapeworms within 32–80 days. Humans are accidental hosts that become infected by ingesting the eggs and, just like the aforementioned herbivorous hosts, allow the development of cysts in various organs. The growth rate of the cysts is about 1 cm per year. The size of the cysts varies between 1 and 15 cm, even though descriptions of cysts of up to 20 cm in diameter can be found in literature. Cysts are typically univesicular, but sometimes small daughter cysts, similar to the mother cyst, can be found in their interior.

Primary skeletal muscle infection with *E. granulosus* accounts for 1%–4% of reported hydatid cases [2]. It may be postulated that the low prevalence of this form of disease is potentially due to the physical barriers to the hematogenous dissemination of cysts created by hepatic sinusoids and pulmonary capillaries. In addition, it has been postulated that the higher lactic acid concentration in skeletal muscle and mechanical factors, such as contractile activity, may make encystment less likely [3]. Nevertheless, some cases of primary muscular hydatidosis at various sites have been reported, that is, biceps brachii [4] thoracic wall [5], sartorius [6, 7], supraspinatus [8], gluteus [9], pterygoideus [10], and soleus muscles [11], whereas only few cases of primary subcutaneous hydatidosis have been reported [12].

Solitary abdominal parietal wall hydatid is a rare finding with only 5 cases reported it is interesting that all five cases reported have hydatid cyst presenting in right iliac region or right paraumbilical region [13–17].

Various pathways have been postulated for involvement of organs other than liver and lung. About 5–15% parasite escape filtering in capillaries in liver and lung to enter systemic circulation to get implanted at various sites, lymphatic spread from intestine to systemic circulation, veno venous shunts in liver, and space of retzius bypassing portal filtering. Waddle [18] proposed an airborne transmission and direct implantation in bronchiole and penetration of bronchial venule to enter left side of heart and systemic circulation. But this remains largely theoretical and needs to be proved.

Probable mechanisms for localization of reported cases of abdominal wall hydatids to right paraumbilical and iliac fossa are direct entry of parasite into inferior vena caval system via connection between systemic veins and portal veins and subsequent reflux implantation of parasites during periods of daily activity associated with Valsalva maneuver. Secondly, penetration of parasites from intestine into peritoneal space and direct invasion of peritoneum in most dependent areas of right paracolic gutter. Thirdly, penetration of parasite into peritoneal lymphatic and localization into abdominal wall.

The clinical course is nonspecific and depends on the site of involvement, the size of the cyst, and the pressure caused by the enlarged cyst. Usually, it presents as an inert, painless, noninflammatory mass without any deterioration of the patient's general condition. However, if superinfected or cracked, the cyst can simulate an abscess or a cancer [19, 20].

Muscular hydatosis resembles a benign neoplasm in many ways. In order to prevent serious complications, it should be diagnosed be made before any therapeutic intervention. The diagnosis is based on the history of exposure in an endemic area and US, CT findings. The diagnosis can be supplemented by specific IgG, complement fixation, indirect fluorescent, and ELISA tests.

Imaging evaluation may be not be specific and accurate and can also indicate other pathological processes, such as malignancy, including sarcoma or infection. Endovesicular daughter cysts that are commonly seen in hepatic hydatid disease imaging are not usually seen on ultrasound or CT of skeletal muscle cysts, and calcification is rare. Ultrasonographical appearances path gnomonic for hydatid cysts include echogenic hydatid sand (the “snowlake sign”), unilocular cysts with daughter cysts “honeycomb sign”, and cysts with a loating detached laminated membrane “waterlily sign”.

 MRI is the examination of choice in case of suspicion of hydatid disease due to its ability to demonstrate adequately most features of hydatid disease, with the exception of calcifications [21]. The multiplanar imaging and the excellent soft tissue contrast provide valuable information on the extent of the disease. The classic MRI indings include a multivesicular cyst, a low-intensity rim “rim sign” on T2-weighted images or a detached membrane [21]. The most pathognomonic sign is that of daughter cysts within larger cysts. According to Díez et al., the presence of viable daughter cysts MRI conveyed as high signal intensity or low signal intensity on T2-weighted images [22]. There is controversy about the value of MRI in diagnosing the vitality of the cyst. Hypointensity of daughter cysts compared with the matrix of the mother cyst on T2-weighted images is a clue for the death of the parasite [23]. Proton density-weighted images generated by gradient echo sequences as a sign of biological activity was suggested by Tekkok et al. [24].

Serology may not always be helpful in diagnosing primary muscle hydatidosis. A negative test does not rule out the diagnosis of echinococcosis. False positivity of Casoni skin test was reported in infestations of tenia saginata and other helminths because of cross reactions. The specificity of Casoni skin test is low because of this high, 40% false positivity. ELISA/Western blood serology is 80–100% sensitive and 88–96% specific for liver cyst infestation, but less sensitive for lung (50–56%) or other organ involvement (25-26%) [25]. Arazi et al. found that 27% (4 of 15) in their case series of musculoskeletal echinococcosis had a positive indirect hemagglutination test [26].

FNAC has proved to be more sensitive and rapid. The hooklets present in necrotic lesions of echinococcus have refractile blades, handles, and guards, and Ziehl-Neelsen stain is particularly useful in identification of the elusive hooklets in necrotic lesions. Hira et al. [27] recommended Wheatly's modifications of Gomori's trichrome stain to identify hooklets. When the laminated membrane fragments are diagnostic, the differential diagnosis is fibrinoid material and mucin (periodic acid-Schiff stain positive) that show pseudolamellar formations. Vercelli-Retta et al. [28] reported that silver methenamine and Best's carmine stain were of special value in identifying laminated layer fragments. Oztek et al. [29] concluded that cytochemical stain and darkfield microscopy are useful in increasing the sensitivity of cytologic detection of hydatid elements (hooklets and laminated membrane fragments) [30]. FNAC of echinococcal cyst in about 1% of cases may cause spillage and anaphylactic shock; use of a small needle is recommended for preventing this.

The treatment of choice in muscular hydatid disease is excision of the intact cyst and surrounding tissue. In one study, cure and mortality rates for the surgical treatment were reported to be >90% and <2%, respectively. Surgical procedures vary from radical procedures (i.e., total cyst excision along with the pericyst) to conservative procedures (i.e., neutralization of the parasite and evacuation of the cyst contents, with the pericyst left in place). Host capsule excision is rarely indicated because the capsule is a part of the host organ and is not infected; and finally, radical procedures require good patient status and surgeon experience [31].

The rationale for albendazole therapy after percutaneous aspiration-injection-reaspiration (PAIR) or surgery is to inactivate viable scoleces in the residual cyst and prevent recurrence. A randomized trial comparing albendazole therapy and PAIR demonstrated maximum reduction in cysts treated with concomitant chemotherapy [32]. Recommendations on the timing of commencement of chemotherapy before surgery or PAIR are varied. Adjunctive chemotherapy initiated before surgery is also of benefit for patients with cysts in inaccessible anatomic locations [33]. The World Health Organization Working Group on Echinococcus recommends preoperative treatment commencing at least 4 days before surgery or PAIR. Factors that should be considered are the procedure to be done and the size and type of cyst. Thick-walled or large cysts may require prolonged treatment before surgery to achieve a scolicidal effect [34]. Viable protoscoleces obtained intraoperatively and after albendazole therapy may be used to predict recurrence [35].

Albendazole has been shown to significantly decrease intracystic pressure when given for 3 weeks before surgery [36]. Exposure time to the drug appears to be more important than the concentrations achieved. This was demonstrated in a prospective randomized study of duration of treatment on cyst viability: after 1 and 3 months of treatment with albendazole for liver cysts, 72% and 94%, respectively, were nonviable [37]. When used alone without surgery, albendazole achieves a cure (cyst disappearance) or improvement, with a reduction in cyst size in up to 50% of cases [38]. Total duration of therapy has varied in studies of PAIR and ranges from 0 to 8 weeks [39, 40].

The benefit of treatment with albendazole after surgery is debatable; treatment with albendazole after surgery is likely to have most benefit in conservative procedures in which there is residual cyst tissue, for cysts in inaccessible anatomic locations, or when there is cyst spillage. The World Health Organization Working Group on Echinococcus recommends 1 month of treatment after surgery. The combination of albendazole and praziquantel has been investigated *in vivo* in a rat model of hydatid infection. In contrast to monotherapy with either agent, combination treatment produced a significant reduction in both the number and viability of cysts [41].

Albendazole is rapidly converted to an active metabolite, albendazole sulfoxide, which achieves high concentrations in the cyst and is active against both protoscoleces and the germinal membranes [42]. Praziquantel does not penetrate into the mature cyst and, therefore, does not inhibit cyst growth, but it is a highly effective protoscolicidal agent both *in vitro* and *in vivo* [43, 44]. The likely role for praziquantel in human hydatidosis may be in preventing encystment of protoscoleces following perioperative spillage [45]. In humans, the use of combination therapy has been reported in the treatment of inoperable spinal, pelvic, abdominal, thoracic, and hepatic hydatidosis [46].

In one study, patients with hydatid cysts were treated with a combination of albendazole and praziquantel. These were compared with historical controls treated with albendazole alone. The complete cure rates were higher in the combined group (47.4% versus 36.4%), although this difference was not statistically significant. In addition, the time to cure was 2–6 months in the combined group, compared with 6–24 months in the albendazole monotherapy group [47]. Therefore, combination therapy may result in a more rapid response than therapy with albendazole alone. Finally, combination therapy was well tolerated, with no increase in adverse events.

## 4. Conclusion

This case illustrates that echinococcal disease should be considered in the differential diagnosis of every cystic mass in any anatomic location, especially when they occur in areas where the disease is endemic. Surgical excision is the treatment of choice with postoperative combined treatment with Albendazole and Praziquantel to prevent recurrence.
